# AI Substitution Perception and Nurses’ Active Learning Behavior: The Mediating Role of Job Insecurity and Moderating Role of Occupational Self‐Efficacy

**DOI:** 10.1155/jonm/5684286

**Published:** 2026-08-23

**Authors:** Yimeng Zheng, Ding Xu, Yongtian Yin, Cuixia Lin, Beibei Liang, Jielong Du

**Affiliations:** ^1^ School of Nursing, Shandong University of Traditional Chinese Medicine, Jinan, Shandong, China, sdutcm.edu.cn; ^2^ School of Health, Shandong University of Traditional Chinese Medicine, Jinan, Shandong, China, sdutcm.edu.cn

**Keywords:** active learning behavior, artificial intelligence technology substitution perception, job insecurity, mediating effect, moderating role, occupational self-efficacy

## Abstract

**Background:**

The rapid advancement of artificial intelligence is profoundly reshaping the nursing profession, potentially raising nurses’ concerns about their professional safety and development, thereby affecting their learning behaviors.

**Aims:**

This study aimed to examine the current state and influencing factors of nurses’ active learning behavior, explore the mediating effect of job insecurity on the relationship between nurses’ artificial intelligence technology substitution perception and active learning behavior, as well as the moderating effect of occupational self‐efficacy.

**Methods:**

Using a convenience sampling method, 560 nurses from 10 hospitals in Shandong Province, China, were recruited from March 1 to March 31, 2026. Data were collected using the General Information Questionnaire, the Perception Scale of AI Technology Substitution, the Job Insecurity Questionnaire, the Occupational Self‐Efficacy Scale‐6, and the Active Learning Behavior Scale. SPSS 26.0 and AMOS 29.0 were used for data analysis.

**Results:**

Nurses’ mean active learning behavior was 23.63 ± 5.59. The direct effect of artificial intelligence technology substitution perception on active learning behavior was 0.232 (46.96%), and the indirect effect via job insecurity was 0.262 (53.04%). Occupational self‐efficacy positively moderated the relationship between job insecurity and active learning behavior (moderated mediation index = 0.007).

**Conclusion:**

The mediating effect of job insecurity between artificial intelligence technology substitution perception and active learning behavior was positively moderated by occupational self‐efficacy.

**Implications for Nursing Management:**

Nursing managers should correctly guide nurses’ perception of artificial intelligence, shape their positive attitude toward artificial intelligence technology, make rational use of job insecurity, and enhance their occupational self‐efficacy, which may help promote nurses’ active learning behavior and career development.

## 1. Introduction

Artificial intelligence (AI) is reconfiguring the healthcare service system with unprecedented depth and breadth [1], and this transformation is profoundly influencing nursing practice. Based on their functional positioning and application scenarios, AI technologies in nursing can be broadly categorized into three types: administrative automation office systems (e.g., intelligent layout [2]), clinical AI collaboration systems (e.g., personalized health management [3]), and medical decision support systems (e.g., AI‐assisted drug decision support [4]). Characterized by high efficiency, accuracy, real‐time monitoring, and objectivity, AI advances the precise, intelligent, streamlined, and collaborative development of nursing practice [5, 6]. Meanwhile, AI has disrupted nurses’ traditional work patterns, requiring them to fully prepare for evolving intelligent technologies and dynamic work environments, with active learning identified as a key strategy for nurses to address this transformation [7]. Active learning behavior stems from nurses’ understanding and forward‐looking judgment of industry developments. It is the process by which nurses, in accordance with their own and their organization’s needs, actively acquire new knowledge and skills and integrate these competencies into clinical practice [8]. Investigating active learning behavior among nurses in the context of intelligent nursing development holds significant theoretical and practical value.

According to a recent report, 39% of existing skills are expected to be transformed or become outdated by 2030, and 40% of employers anticipate reducing their workforce due to skill mismatches [9]. The 2026 AI Index Report further reveals that 52% of the public express concerns rather than excitement about AI [10]. This trend is also reflected in nursing. As users of AI technology, nurses, while benefiting from technological empowerment, simultaneously experience concerns and anxieties about career stability, role value, and development prospects due to the proliferation of AI [11, 12], creating a paradoxical perception of technological empowerment versus career threats, which is defined as the AI technology substitution perception. From the perspective of professional identity, AI empowerment may encourage nurses to conduct in‐depth reflections on the professionalism, social role, and scope of duties [13]. When AI can partially undertake tasks such as disease monitoring and nursing documentation, nurses may worry about restrictions on their independent decision‐making and the weakening of their professional skills, resulting in confusion over their core values and career positioning [14]. The negative perceptions induced about by technological changes can trigger anxiety among nurses, affecting their willingness to adopt new technologies and hindering their innovative behaviors [11]. Furthermore, the disruptive impact of AI can also heighten individuals’ work vigilance, enhance their competitive awareness, strengthen their learning motivation, and form internal drive [15]. Currently, numerous studies have examined AI‐driven technology change and employee initiative behavior, but most of these studies have focused on the fields of business management and education [15, 16]. Studies in nursing have mostly focused directly on factors such as nurses’ acceptance of AI, emotional responses, or willingness to use it [17]. There remains insufficient exploration of the underlying pathways through which the AI technology substitution perception affects nurses’ active learning behavior via psychological mechanisms.

According to the Conservation of Resources theory (COR), resource loss threats trigger stress responses in individuals [18]. This risk judgment of potential future resource depletion may trigger actual behaviors through psychological state of job insecurity. Job insecurity in the AI era is conceptualized as concerns about job replacement due to AI, as well as the sense of incompetence experienced when employees update their skills to achieve effective human–machine collaboration [19]. Previous studies have confirmed that the sense of substitution induced by AI and the awareness of technological impact can lead to job insecurity among employees [20]. Such job insecurity is closely related to positive behaviors such as active learning and innovative behavior [21]. The job insecurity brought by AI will prompt employees to reevaluate their own resource endowments and accordingly form corresponding solutions. This indicates that when nurses experience job insecurity, they may tend to view knowledge and skills as key resources for investment and accumulation [22]. This cognition may prompt individuals to enhance their resource reserves through active learning to cope with potential career risks and avoid resource depletion caused by AI substitution perception.

The COR theory posits that individual differences in resource reserves are associated with cognitive appraisals of resource threats and coping strategies. Individuals with higher resource reserves can not only effectively avoid the risk of resource loss but also demonstrate stronger resource appreciation capabilities [23]. Occupational self‐efficacy is the subjective perception formed by an individual after judging and evaluating their own ability to perform professional tasks [24]. As a specific manifestation of self‐efficacy in the occupational domain, it is a crucial internal resource for individuals [25]. In the context of rapidly changing workplace technologies, occupational self‐efficacy is particularly crucial, as it reflects whether an individual has the confidence to acquire new skills and adapt to new roles [26]. Research has confirmed that occupational self‐efficacy is related to nurses’ workplace learning potential [24]. This implies that when nurses perceive job insecurity caused by technological changes, those with high occupational self‐efficacy may tend to regard it as an opportunity for self‐improvement and demonstrate greater learning abilities and initiative.

This study, guided by the COR, adopted a cross‐sectional design to explore the underlying mechanism of nurses’ AI technology substitution perception and active learning behavior. The AI technology substitution perception was used as the independent variable, representing the resource loss perceived by nurses, and might be directly related to nurses’ active learning behavior. Job insecurity served as the mediating variable, linking perception of technical threat and nurses’ learning behavior. Occupational self‐efficacy, as the cognitive moderating variable, might be related to the coping strategies that nurses adopt toward resource threats. We proposed the following hypotheses:

 H1: The AI technology substitution perception is positively associated with active learning behavior;
 H2: The AI technology substitution perception has a positive indirect association with active learning behavior through the mediating effect of job insecurity;
 H3: Occupational self‐efficacy plays a positive moderating role in the relationship between job insecurity and active learning behavior.

## 2. Methods

### 2.1. Study Design

Using a convenience sampling method, from March 1 to March 31, 2026, nurses from 10 hospitals in Shandong Province, China, were recruited for the survey. The measurement model consisted of 17 observed variables. A minimum of 208 participants was required for the structural equation models, as determined by G∗Power 3.1 with *f* ^2^ = 0.15, α = 0.05 and power 1−*β* = 0.95. This study collected 581 questionnaires. After eliminating 21 invalid questionnaires, 560 eligible questionnaires were retained, with a valid response rate of 96.39%.

### 2.2. Inclusion and Exclusion Criteria

Inclusion criteria were as follows: (1) have obtained the nurse qualification certificate and completed the registration; (2) clinical nursing work for no less than 6 months; (3) informed consent and voluntary participation. Exclusion criteria were as follows: (1) nonclinical positions; (2) those on temporary absence during the study period.

### 2.3. Instruments

1. General Information Questionnaire
 A self‐developed questionnaire collected demographic data, including gender, age, professional title, average monthly income, etc.
2. Perception Scale of Artificial Intelligence Technology Substitution (PSAT)
 This scale was developed by Brougham and Haar [27] in 2018 and was subsequently validated and applied by Fan et al. [11] among Chinese nurses to assess individuals’ AI technology substitution perception. The unidimensional 4‐item scale uses a 7‐point Likert format (1 = *strongly disagree* to 7 = *strongly agree*). Higher scores indicate greater AI technology substitution perception. The Chinese version of the scale exhibited good reliability, with a Cronbach’s *α* coefficient of 0.782 in this study.
3. Job Insecurity Questionnaire (JIQ)
 This questionnaire was developed by Hu et al. [28] in 2009 to assess individual’s level of job insecurity. It consists of 9 items covering two dimensions: quality job insecurity (5 items) and quantity job insecurity (4 items). The scale uses a 7‐point format ranging from 1 (“*very inconsistent*”) to 7 (“*very consistent*”), with higher scores indicating greater job insecurity. The Cronbach’s *α* coefficient of the original questionnaire was 0.922. In this study, the Cronbach’s *α* coefficient of the questionnaire was 0.882.
4. Occupational Self‐Efficacy Scale‐6 (OSS‐6)
 This scale was developed by Rigotti et al. [29] in 2008 to assess individual’s occupational self‐efficacy. The scale is unidimensional with 6 items. Items were rated on a 5‐point Likert scale ranging from 1 (“*completely disagree*”) to 5 (“*completely agree*”). Higher scores indicate greater occupational self‐efficacy. Previous studies [30] in China have verified that this scale has good reliability and validity. In this study, the Cronbach’s *α* coefficient was 0.897.
5. Active Learning Behavior Scale (ALB)
 This scale was developed by Bezuijen et al. [31] in 2010 to measure individual’s active learning behavior. Rated on a 5‐point Likert scale (1 = *completely disagree* to 5 = *completely agree*), this 8‐item scale produces higher scores that denote more active learning behavior. The Cronbach’s *α* coefficient of the original scale ranged from 0.78 to 0.88. This study adopted the Chinese version translated, cross‐culturally adapted, and revised by Zhu and Wu [32]. In this study, the Cronbach’s *α* coefficient of this scale was 0.880.

All the scales used in this study received noncommercial research use authorization from the original authors or the Chinese version revisers. SPSS 26.0 was used to evaluate the reliability of all scales. The use of the scales followed academic norms and was solely for the purpose of this study.

### 2.4. Data Collection

This study was conducted through online questionnaires. Standardized instructions explained the study aim and significance. All questionnaires were collected anonymously. Nurses completed the questions under the conditions of informed consent. All items were required, and each participant could submit the questionnaire only once. Questionnaires with patterned responses, a completion time of less than 120 s, or logical inconsistencies were eliminated.

### 2.5. Statistical Analysis

SPSS 26.0 and AMOS 29.0 were used for data analysis. General data were analyzed using descriptive statistics. Group comparisons were performed using *t*‐tests, rank sum tests, and other relevant tests, while correlations were analyzed via Spearman correlation analysis. Common method bias was tested using confirmatory factor analysis (CFA). Multiple linear regression was used to analyze the influencing factors of nurses’ active learning behavior. The model was verified using CFA. The mediating effect was tested using 5000 bootstrap repeated sampling. The test level *α* = 0.05.

### 2.6. Ethical Considerations

The study was approved by the Jinan Maternity and Child Care Hospital Research Ethics Committee (approval number: KY R‐25‐096).

## 3. Results

### 3.1. Sample Characteristics

Among all participants, 14.6% were male, while 85.4% were female. Most of the nurses were aged 18–30 years (72.1%), unmarried (57.5%), held bachelor degree (69.1%), had 6‐10 years of working experience (45.5%), were junior nurse (72.1%), earned an average monthly income of 5000–10,000 yuan (65.7%), were specialist nurses (82.0%), had received AI training (79.8%), and had used AI equipment (90.4%) (Table 1).

**TABLE 1:** General characteristics.

Variables	Category	*n*	%
Gender	Male	82	14.6
	Female	478	85.4

Age	18–30	404	72.1
	31–45	152	27.1
	≥ 46	4	0.7

Marital status	Unmarried	322	57.5
	Married	222	39.6
	Widowed	10	1.8
	Divorced	6	1.1

Educational level	Junior college or below	154	27.5
	Bachelor	387	69.1
	Master degree or above	19	3.4

Years of work experience	≤ 5	248	44.3
	6–10	255	45.5
	11–15	48	8.6
	16–20	9	1.6

Professional title	Junior nurse	404	72.1
	Intermediate nurse	151	27.0
	Senior nurse	5	0.9

Average monthly income	< 5000	164	29.3
	5000–10,000	368	65.7
	> 10,000	28	5.0

Specialist nurses	No	101	18.0
	Yes	459	82.0

AI training	No	113	20.2
	Yes	447	79.8

Used AI equipment	No	54	9.6
	Yes	506	90.4

### 3.2. Descriptive Statistics and Bivariate Correlations of Nurses’ AI Technology Substitution Perception, Job Insecurity, Occupational Self‐Efficacy, and Active Learning Behavior

Mean scores were 14.95 ± 5.72 for AI technology substitution perception, 34.16 ± 12.17 for job insecurity, 20.02 ± 5.59 for occupational self‐efficacy, and 23.63 ± 5.59 for active learning behavior (Table 2). Spearman correlation analysis showed that AI technology substitution perception, job insecurity, and occupational self‐efficacy were positively correlated with active learning behavior (*p* < 0.01) (Table 3).

**TABLE 2:** Nurses’ AI technology substitution perception, job insecurity, occupational self‐efficacy, and active learning behavior scores.

Variables	Min	Max	M	SD
PSAT	4	26	14.95	5.72
JIQ	9	58	34.16	12.17
Quality job insecurity	5	33	18.91	7.00
Quantity job insecurity	4	27	15.25	5.87
OSS‐6	6	29	20.02	5.59
ALB	8	38	23.63	5.59

*Note:* PSAT, Perception Scale of Artificial Intelligence Technology Substitution; OSS‐6, Occupational Self‐Efficacy Scale‐6; Min, minimum; Max, maximum; M, mean.

Abbreviations: ALB, active learning behavior; JIQ, job insecurity questionnaire; SD, standard deviation.

**TABLE 3:** Correlation analysis of nurses’ active learning behavior with AI technology substitution perception, job insecurity, and occupational self‐efficacy.

Variables	ALB	PSAT	JIQ	Quality job insecurity	Quantity job insecurity	OSS‐6
ALB	1.000					
PSAT	0.834^∗∗^	1.000				
JIQ	0.876^∗∗^	0.797^∗∗^	1.000			
Quality job insecurity	0.839^∗∗^	0.757^∗∗^	0.952^∗∗^	1.000		
Quantity job insecurity	0.801^∗∗^	0.737^∗∗^	0.924^∗∗^	0.770^∗∗^	1.000	
OSS‐6	0.852^∗∗^	0.730^∗∗^	0.795^∗∗^	0.772^∗∗^	0.718^∗∗^	1.000

*Note:* PSAT, Perception Scale of Artificial Intelligence Technology Substitution; OSS‐6, Occupational Self‐Efficacy Scale‐6.

Abbreviations: ALB, active learning behavior; JIQ, job insecurity questionnaire.

^∗∗^ *p* < 0.01.

### 3.3. Univariate Analysis of Nurses’ Active Learning Behavior

The results showed that age, educational level, years of work experience, professional title, average monthly income, AI training, and use of AI equipment had statistically significant associations with nurses’ active learning behavior (*p* < 0.05) (Table 4).

**TABLE 4:** Univariate analysis of active learning behavior.

Variables	Category	ALB	U/H	*p*
Gender	Male	24.54 ± 5.36	−1.909	0.056
	Female	23.47 ± 5.61		

Age	18–30	23.04 ± 5.50	26.702	< 0.001
	31–45	25.05 ± 5.53		
	≥ 46	28.75 ± 5.25		

Marital status	Unmarried	23.73 ± 5.35	5.707	0.127
	Married	23.25 ± 5.98		
	Widowed	26.40 ± 3.86		
	Divorced	27.33 ± 2.66		

Educational level	Junior college or below	25.90 ± 4.26	43.738	< 0.001
	Bachelor	22.79 ± 5.81		
	Master degree or above	22.32 ± 5.48		

Years of work experience	≤ 5	24.11 ± 5.50	25.528	< 0.001
	6–10	22.60 ± 5.58		
	11–15	25.73 ± 5.08		
	16–20	28.11 ± 4.17		

Professional title	Junior nurse	24.03 ± 5.57	10.567	0.005
	Intermediate nurse	22.46 ± 5.48		
	Senior nurse	26.20 ± 5.40		

Average monthly income	< 5000	24.65 ± 5.53	10.078	0.006
	5000–10,000	23.29 ± 5.57		
	> 10,000	22.11 ± 5.38		

Specialist nurses	No	23.29 ± 5.99	−0.860	0.390
	Yes	23.70 ± 5.50		

AI training	No	27.02 ± 3.48	−7.458	< 0.001
	Yes	22.77 ± 5.70		

Used AI equipment	No	25.91 ± 5.13	−3.103	0.002
	Yes	23.38 ± 5.58		

Abbreviation: ALB, active learning behavior.

### 3.4. Common Method Bias Test

Model fit indices of the single‐factor CFA model were *χ* ^2^ */*df = 5.539, RMSEA = 0.090, CFI = 0.992, TLI = 0.983, indicating poor model fit and the absence of serious common method bias in this study. Collinearity diagnosis revealed that the variance inflation factor values for all independent variables were < 5, suggesting no severe multicollinearity.

### 3.5. Multiple Regression Analysis of Nurses’ Active Learning Behavior Predictors

Taking the total score of nurses’ active learning behavior as the dependent variable, and including the variables with statistical significance from the univariate factor analysis, AI technology substitution perception, job insecurity, and occupational self‐efficacy as independent variables, a multiple regression analysis was conducted. The results showed that educational level, AI technology substitution perception, job insecurity, and occupational self‐efficacy were significant predictors of nurses’ active learning behavior (*p* < 0.05) (Table 5).

**TABLE 5:** Multiple regression analysis of factors influencing nurses’ active learning behavior.

Variables	*B*	SE	Beta	*t*	*p*	95% CI
Constant	6.828	0.633		10.786	< 0.001	5.584–8.071
Age						
18–30 (reference)						
31–45	0.420	0.307	0.033	1.368	0.172	−0.183–1.023
≥ 46	1.816	1.776	0.027	1.023	0.307	−1.673–5.305
Educational level						
Junior college or below (reference)						
Bachelor	0.546	0.259	0.045	2.106	0.036	0.037–1.056
Master degree or above	1.639	0.652	0.053	2.515	0.012	0.359–2.919
Years of work experience						
≤ 5 (reference)						
6–10	−0.124	0.289	−0.011	−0.429	0.668	−0.692–0.444
11–15	−0.100	0.510	−0.005	−0.196	0.845	−1.101–0.902
16–20	0.714	1.159	0.016	0.616	0.538	−1.563–2.992
Professional title						
Junior nurse (reference)						
Intermediate nurse	−0.370	0.296	−0.029	−1.251	0.211	−0.951–0.211
Senior nurse	−0.414	1.478	−0.007	−0.280	0.780	−3.317–2.490
Average monthly income						
< 5000 (reference)						
5000–10,000	−0.183	0.247	−0.016	−0.738	0.461	−0.669–0.303
> 10,000	−0.209	0.582	−0.008	−0.359	0.720	−1.352–0.934
AI training	−0.232	0.285	−0.017	−0.815	0.415	−0.792–0.328
Used AI equipment	0.280	0.354	0.015	0.789	0.430	−0.416–0.975
PSAT	0.233	0.030	0.239	7.796	< 0.001	0.175–0.292
JIQ	0.165	0.017	0.360	9.474	< 0.001	0.131–0.199
OSS‐6	0.366	0.032	0.379	11.470	< 0.001	0.303–0.429

*Note:* PSAT, Perception Scale of Artificial Intelligence Technology Substitution; OSS‐6, Occupational Self‐Efficacy Scale‐6. *R* ^2^ = 0.836, adjusted *R* ^2^ = 0.831, *F* = 172.452, DW = 1.860, *p* < 0.001.

Abbreviations: CI, confidence interval; JIQ, job insecurity questionnaire.

### 3.6. Hypothesis Testing of Nurses’ Active Learning Behavior

CFA demonstrated that the four‐factor model yielded the best fit to the data, as shown in Table 6. Moreover, the CR (> 0.7) and AVE (> 0.5) for each dimension reached the thresholds, and the square root of AVE was also greater than the cross‐dimensional correlation coefficients, indicating that the scales demonstrated adequate convergent validity and discriminant validity. The results of model fit and validity tests jointly indicated that common method bias did not significantly interfere with the results of this study.

**TABLE 6:** Model fit comparison.

Measurement model	*χ* ^2^ */*df	RMSEA	SRMR	CFI	TLI
Single factor (PSAT + JIQ + OSS‐6 + ALB)	5.539	0.090	0.0114	0.992	0.983
Two factors (PSAT + ALB, JIQ + OSS‐6)	3.547	0.067	0.0082	0.996	0.991
Three factors (PSAT, JIQ, OSS‐6 + ALB)	3.208	0.063	0.0075	0.998	0.992
Four factors (PSAT, JIQ, OSS‐6, ALB)	2.087	0.044	0.0051	0.999	0.996

*Note:* PSAT, Perception Scale of Artificial Intelligence Technology Substitution; OSS‐6, Occupational Self‐Efficacy Scale‐6.

Abbreviations: ALB, active learning behavior; JIQ, job insecurity questionnaire.

**FIGURE 1:**
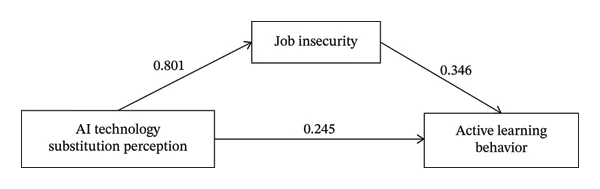
Mediation model diagram of nurses’ job insecurity between AI technology substitution perception and active learning behavior.

In the mediation model, AI technology substitution perception was the independent variable, job insecurity was the mediating variable, and active learning behavior was the dependent variable. As shown in Figure 1, the analysis showed that AI technology substitution perception had a direct effect on active learning behavior, with an effect size of 0.232, accounting for 46.96% of the total effect. Job insecurity exerted an indirect effect between AI technology substitution perception and active learning behavior, with an effect size of 0.262, accounting for 53.04% of the total effect, *p* < 0.001 (Tables 7 and 8).

**TABLE 7:** Path coefficient test.

Path	Estimate	S.E.	C.R.	*p*
PSAT ⟶ JIQ	0.801	0.054	31.660	0.001
PSAT ⟶ ALB	0.245	0.035	6.549	0.001
JIQ ⟶ ALB	0.346	0.013	11.547	0.001

*Note:* PSAT, Perception Scale of Artificial Intelligence Technology Substitution.

Abbreviations: ALB, active learning behavior; JIQ, job insecurity questionnaire.

**TABLE 8:** Analysis of the mediating effect of nurses’ job insecurity between AI technology substitution perception and active learning behavior.

Path	Estimate	SE	95% CI	*p*	Effect ratio (%)
Direct effect	0.232	0.039	0.161–0.311	< 0.001	46.96
Indirect effect	0.262	0.037	0.196–0.342	< 0.001	53.04
Total effect	0.494	0.047	0.407–0.588	< 0.001	100

Abbreviations: CI, confidence interval; SE, standard error.

This study used the Bootstrap method to test the moderating effect of occupational self‐efficacy. The results showed that, compared with the low occupational self‐efficacy level, the indirect effect of job insecurity on active learning behavior was stronger under the high occupational self‐efficacy level, and the difference between the two impact coefficients was significant (*β* = 0.045, *p* < 0.05), as detailed in Table 9. The moderated mediation index was 0.007. This indicated that occupational self‐efficacy positively moderated the indirect effect of AI technology substitution perception on the active learning behavior through job insecurity, and the moderated mediation effect was significant.

**TABLE 9:** Moderated mediation tests.

Mediating variable	Moderating variable	Estimate	SE	95% CI	*p*
JIQ	Low level (−1SD)	0.131	0.025	0.081–0.180	< 0.001
	High level (+1SD)	0.176	0.020	0.139–0.219	< 0.001
	Intergroup differences (High‐Low)	0.045	0.023	0.002–0.092	0.040

Abbreviations: CI, confidence interval; JIQ, job insecurity questionnaire; SE, standard error.

## 4. Discussion

### 4.1. Current Status and Influencing Factors of Clinical Nurses’ Active Learning Behavior

In this study, the total score of nurses’ active learning behavior was 23.63 ± 5.59, with a mean score of 2.95 ± 0.70 (out of 5), indicating a moderate level. This indicates that although nurses have initiated learning responses to the rapid penetration of AI into practice, considerable room remains for improving their overall initiative. After adjusting for other covariates, multivariate analysis identified educational level, in addition to the three core variables of AI technology substitution perception, job insecurity, and occupational self‐efficacy, as an independent predictor of active learning behavior. Nurses with higher educational level tended to have higher active learning behavior scores. This may be attributed to the self‐learning ability, critical thinking, higher intrinsic motivation, and career development expectations fostered by nurses’ advanced education [33]. Research has found that nurses with higher educational levels exhibit higher levels of awareness and proactive work adjustment behaviors [34]. When faced with occupational risks and job insecurity induced by AI technology substitution, they can engage in systematic and in‐depth self‐learning to consolidate their professional resources and competitive advantages.

Furthermore, the variables in this study showed high positive correlations. However, statistical tests indicated that common method bias and discriminant validity were within acceptable ranges. This might partly reflect the coordinated changes in nurses’ stress‐coping systems amid the transformation of AI technology: AI technology substitution, as a potential threat of resource loss, triggered nurses’ cognitive appraisals, motivation activation, and resource beliefs. These reactions collectively formed an interrelated and collaboratively changing stress‐coping system [35], thus potentially presenting a high degree of correlation.

### 4.2. Nurses’ AI Technology Substitution Perception Positively Correlated With Active Learning Behavior

This study showed that nurses’ AI technology substitution perception was positively correlated with active learning behavior. The higher the AI substitution perception among nurses, the more likely they were to actively engage in professional learning and technical training. This result differed from previous studies on the negative behaviors caused by technological pressure [11, 36]. The positive finding of this study suggests that AI technology substitution might not be solely a source of negative stress. According to the resource investment principle of COR theory [18], when nurses intuitively sense that AI technology may change clinical work structure, affect salary and remuneration, and even replace job roles [37], in order to avoid falling into a resource loss spiral and triggering a cascade of further losses, nurses may adopt resource investment behaviors such as active learning to increase their knowledge reserves and consolidate their professional competitiveness. Through long‐term nursing practice, nurses have gained an in‐depth understanding of the functions and limitations of AI [38]. They realize that AI can replace nurses in performing nursing tasks, but it lacks the ability for caregiving and ethical reasoning, and thus cannot truly replace nurses [39]. This understanding has shifted the role positioning of nurses from technical adopters to system controllers and ethical supervisors [38, 40], which might be associated with an increased internal driving force for active learning. To fulfill responsibilities such as reviewing AI algorithms, overseeing clinical decisions, safeguarding patients’ rights, and maintaining the ethical boundaries of human–machine collaboration, nurses need to enhance their own competencies to meet the role requirements for AI usage and supervision [41]. Therefore, in this research context, the “threat–motivation–learning” pathway prevails, which may be related to nurses’ AI substitution perception as a sign of role advancement.

### 4.3. The Mediating Effect of Job Insecurity Between Nurses’ AI Technology Substitution Perception and Active Learning Behavior

This study found that job insecurity played a partial mediating role between nurses’ AI technology substitution perception and active learning behavior, accounting for 53.04% of the total effect. The COR theory [18] states that psychological stress stems from the subjective perception of expected resource loss. The loss priority principle emphasizes that resource loss outweighs gain in impact, so individuals strive to take actions to prevent the occurrence of resource loss [42]. The introduction of AI in the nursing field may lead nurses to worry about job displacement, weakened nurse–patient communication, and ambiguous legal boundaries, which constitute the main sources of nurses’ job insecurity [43]. When this insecurity is interpreted by nurses as a challenge, it may stimulate their proactive coping behaviors [44]. Of note, the job insecurity–active behavior relationship may be nonlinear. A previous study suggested that moderate job insecurity was positively correlated with employees’ active behavior, while lower or higher levels of job insecurity might inhibit employees’ innovative and other active behaviors [45]. In this study, the level of job insecurity among nurses was found to be moderate, which corroborated previous findings, suggesting that the relationship between job insecurity and behavior might have a threshold effect.

### 4.4. Occupational Self‐Efficacy Played a Positive Moderating Role Between Nurses’ Job Insecurity and Active Learning Behavior

This study found that occupational self‐efficacy positively moderated the relationship between nurses’ job insecurity and active learning behavior. When occupational self‐efficacy was high, the positive correlation between job insecurity and active learning behavior was stronger; under low self‐efficacy conditions, this association was weakened. Studies have demonstrated that self‐efficacy is positively correlated with nurses’ learning behavior, professional adaptability [24, 46], and attitudes toward technological changes, which may help nurses cope with the professional challenges arising from AI [47]. From the perspective of COR, resource availability is closely related to the strategies individuals adopt to cope with stress. Self‐efficacy, as a form of personal resource [18], reflects nurses’ belief in their own coping abilities. Nurses who possess occupational self‐efficacy may view technological changes as both challenges and opportunities [48], and tend to adopt proactive coping strategies to deal with the resulting job insecurity, such as actively engaging with AI technology. Conversely, nurses with low occupational self‐efficacy may, due to a lack of professional confidence, be more likely to adopt defensive behaviors such as reducing work input and resisting the application of AI when facing the same level of job insecurity. These defensive responses, to some extent, may inhibit the transformation from job insecurity to active learning behavior [42]. The primary mechanism through which occupational self‐efficacy functions may be to enhance coping responses: it improves the psychological readiness and execution abilities of nurses to transform insecurity into specific learning actions. Yao et al. [48] showed that self‐efficacy could moderate the association between job insecurity and active behavior. This finding to some extent supports the view of this study. Therefore, boosting nurses’ occupational self‐efficacy may strengthen their learning adaptability in the context of technological changes.

These findings suggested that nurse managers may emphasize the clinical support function of AI through thematic presentations and case sharing. Meanwhile, job insecurity can be regulated at a manageable level by appropriately allocating workloads and conducting periodic psychological evaluations. In addition, occupational self‐efficacy may be strengthened via skills training and performance feedback. Collectively, these strategies can help nurses transform technological uncertainty into motivation for active learning, thereby enhancing their adaptability to ongoing digital transformation in nursing.

## 5. Limitations

Several limitations must be noted. First, the cross‐sectional design cannot directly verify causality. Future research can employ mixed research methods, such as qualitative research and longitudinal study designs. Second, all participants were recruited solely from Shandong Province, China, limiting the findings’ generalizability. Future research can conduct multicenter, large‐sample investigations to improve the generalizability of the results. Third, all measurement instruments in this study were self‐reported scales, which may introduce response biases. Moreover, the high intercorrelations between variables suggest that there may exist a certain degree of ambiguity in their conceptual boundaries. Future research can use multisource data or multiple methods for more rigorous testing. Fourth, nurses’ active learning behavior in the AI context is shaped by multiple factors. This study failed to consider multiple critical organizational factors that may influence nurses’ active learning behavior, including transformational leadership, human resource allocation, culture and policy, organizational support, and learning goal orientation. Future research can incorporate these factors for more comprehensive analytical results.

## 6. Conclusion

This study found that AI technology substitution perception was directly and positively associated with nurses’ active learning behaviors. Job insecurity partially mediated this relationship, whereas occupational self‐efficacy positively moderated this mediating pathway. These findings extend the COR theory, showing how resource loss appraisal, psychological states, and personal resources may jointly relate to nurses’ active learning behaviors in the context of nursing AI transformation. This study provides empirical evidence for nursing managers to facilitate nurses’ proactive learning amid AI‐driven industrial transformation.

## Author Contributions

All authors contributed to the study design. Yimeng Zheng drafted, revised, and reviewed the manuscript. Ding Xu, Yimeng Zheng, Beibei Liang, and Jielong Du contributed to the data collection. Ding Xu conducted the data analysis and revised and reviewed the manuscript. Cuixia Lin was involved in the study supervision. Yongtian Yin provided study supervision.

Yimeng Zheng and Ding Xu are the co‐first authors. Yongtian Yin is the corresponding author, E‐mail: yinyongtian2004@163.com.

## Funding

No funding was received for this research.

## Conflicts of Interest

The authors declare no conflicts of interest.

## Data Availability

The data that support the findings of this study are available on request from the corresponding author. The data are not publicly available due to privacy or ethical restrictions.
